# Syndrome d’activation macrophagique révélant un lymphome T sous-cutané chez un adolescent de 16 ans

**DOI:** 10.11604/pamj.2018.31.74.14686

**Published:** 2018-10-02

**Authors:** Mohamed Hbibi, Sara Benmiloud, Safae Rahmouni, Ilhame Tadmouri, Sana Abourazzak, Sana Chaouki, Fatima Zahra Souilmi, Mounia Lakhdar Idrissi, Mostapha Hida

**Affiliations:** 1 Faculté de Médecine et de Pharmacie de Fès, Université Sidi Mohamed Ben Abdellah, Service de Pédiatrie CHU Hassan II, Fès, Maroc

**Keywords:** Lymphome T sous-cutanée, syndrome d´activation macrophagique, panniculite, Subcutaneous T-cell lymphoma, macrophage activation syndrome, panniculitis

## Abstract

Le syndrome d'activation macrophagique (SAM) est une atteinte multisystémique, liée à une intense activation du système immunitaire correspondant à une infiltration plus ou moins diffuse des tissus par des macrophages activés. Il associé des signes cliniques (fièvre, hépato splénomégalie, adénopathie) et des anomalies biologiques (bi ou pancytopénie, cytolyse hépatique, élévation des LDH, coagulopathie) à une hémophagocytose .Il peut être primaire chez l'enfant ou secondaire à diverses affections. Nous rapportons le cas d'un adolescent de 16 ans admis au service de pédiatrie pour leucopénie fébrile avec altération de l'état général et des lésions érythémateuses circulaires étendues au niveau des membres inférieurs. Le diagnostic de syndrome d'activation macrophagique était retenu devant les signes cliniques, biologiques et cytologiques compatibles. La biopsie cutanée de ces lésions était en faveur d'un lymphome T sous-cutané type panniculite. A travers ce travail nous insistons sur la particularité de cette observation clinique vu la rareté de ce type de lymphome T sous cutanée et beaucoup plus au cours de cette tranche d'âge, ainsi sur l'intérêt de penser au lymphome T sous cutanée devant un SAM qui pourrait mettre en jeu le pronostic vital, lorsque il est associé à des lésions sous cutanée érythémateuses.

## Abstract (en)

Macrophage activation syndrome (MAS) is a multisystemic disorder resulting from an over-activation of the immune system leading to a more or less diffuse macrophagic infiltration into the tussues. Clinical signs including fever, hepatosplenomegaly, adenopathy are associated with abnormal values in laboratory test results (bi or pancytopenia, hepatic cytolysis, elevated LDH levels, coagulopathy) and hemophagocytosis. In children, it can be primary or secondary to several disorders. We report the case of a 16-year old patient admitted to the Department of Pediatrics with febrile leukopenia associated with impaired general condition and circular erythematous lesions spread to the lower limbs. The diagnosis of macrophage activation syndrome was retained based on clinical signs, laboratory test results and cytological results. Skin biopsy of these lesions showed subcutaneous panniculitis-like T-cell lymphoma. The purpose of this study is to insists on the peculiarities of this clinical case given the rarity of this type of subcutaneous T-cell lymphoma, much more in this age group. We want to highlight the importance of suspecting subcutaneous T-cell lymphoma in patients with MAS associated with subcutaneous erythematous lesions because this could be life threatening.

## Introduction

Le syndrome d'activation macrophagique (SAM) est un syndrome grave potentiellement mortel due à une activation immunitaire excessive [1, 2]. Il affecte le plus souvent les nourrissons, mais aussi il est observé chez les enfants et les adultes à tout âge [3]. Le SAM peut être primaire ou secondaire à diverses affections [1, 3]. Dans ce cas clinique, le SAM révèle un lymphome T sous-cutanée type panniculite qui est une entité rare des lymphomes cutanés. L'association de SAM et d'un lymphome T sous-cutané est décrite mais rare et touche généralement l'adulte [4].

## Patient et observation

**Figure 1:**
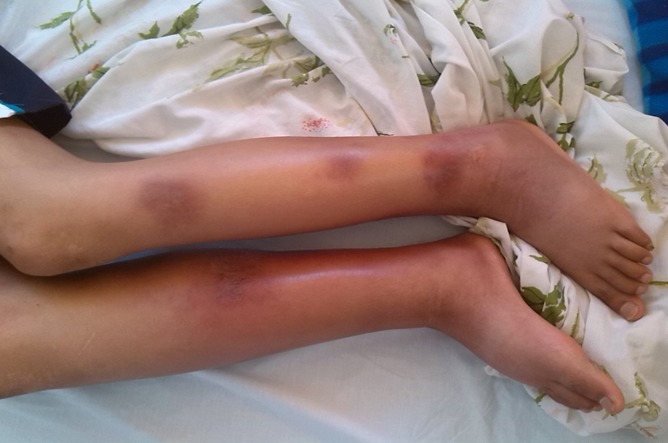
Lésions érythémateuses en placard infiltré et induré inflammatoires non douloureuses

Un adolescent de 16 ans était adressé pour une leucopénie fébrile associée à une hypodermite. Dans ses antécédents on notait une pelade à l'âge de 6 ans avec notion d'application d'un traitement traditionnel et des médicaments non documentés. Deux mois avant son hospitalisation étaient apparus des lésions érythémateuses en placard infiltrées et indurées inflammatoires non douloureuses siégeant au niveau des cuisses. L'apparition secondaire d'arthralgies des genoux et l'aggravation de l'état général avec fièvre motivaient son hospitalisation. A l'examen, un mauvais état général était constaté, la température était à 39°C. Il avait un poids de 50 kg pour une taille de 176 cm. L'examen cutané objectivait des lésions érythémateuses en placard infiltré et induré inflammatoires non douloureuses siégeant au niveau de la face antéro-interne et postérieur des cuisses, avec des lésions circulaires au niveau des jambes, on notait également des lésions papuleuses et inflammatoires du dos et du tronc, ainsi que plusieurs plaques de pelades (Figure 1). Il n'y avait pas d'adénopathie palpable ni d'hépatomégalie ou splénomégalie. Sur le plan biologique, il existait un syndrome inflammatoire modéré avec une protéine C réactive à 72 mg/l et une vitesse de sédimentation à la première heure: 35 mm et 90 mm à la deuxième heure. L'hémogramme montrait une bi-cytopénie: Hb: 10,2 g/dl, VGM: 80,1, CCMH: 32,6, GB: 1790/mm^3^, PNN: 1030/mm^3^, lymphocytes: 630/mm^3^, plaquettes: 250000/mm^3^. Les enzymes hépatiques étaient élevés GOT:105 UI, GPT: 70 UI. L'électrophorèse des protéines montrait une hypoalbuminémie associé à une augmentation en alpha 1 globuline. Les triglycérides étaient à 1,66g/l. La ferritine était très élevée a 1992. Les lacticodéshydrogénases LDH étaient augmentées à 1156. Les anticorps anti-DNA et antinucléaires étaient négatives, les sérologies virales HIV, hépatite A, B, C aussi négatives. Le médullogramme était en faveur d'une hémophagocytose. Ce bilan est en faveur d'un SAM. Par ailleurs la biopsie cutanée montrait un aspect morphologique et immunohistochimique en faveur d'un lymphome T sous cutané (CD8+, CD4-, CD56-). l'évolution au cours de son séjour était marquée par l'altération de l'état général avec persistance de la fièvre, et le malade avait reçu des bolus de corticoïdes vu le SAM avec une amélioration de son état, puis fut transféré au service de médecine interne pour chimiothérapie.

## Discussion

Dans cette observation, un lymphome T sous cutané a été découvert chez un adolescent au cours d'une altération de l'état générale avec un syndrome d'activation macrophagique (SAM).Le diagnostic a été posé par l'examen histologique et immunohistochimique d'une biopsie cutanée. Le SAM est une pathologie rare et grave [5] dont la mortalité peut atteindre 50% des cas. Il est lié à une activation inappropriée du système immunitaire, dont la traduction est une infiltration tissulaire par les macrophage activés [1, 3]. Le diagnostic est basé sur l'association des signes cliniques et biologiques non spécifiques, imposant la recherche cytologique d'une hémophagocytose [1, 3, 6] et d'une enquête étiologique assez exhaussive.Il peut être primaire ou secondaire à diverses affections [3, 6]. Dans notre cas, le SAM est secondaire à un lymphome T sous cutané type panniculite. Ce dernier est une entité rare [7] au sein d'un groupe hétérogène des lymphomes cutanés, classé dans la nouvelle classification WHO-EORTC [8]. Ce lymphome décrit pour la première fois en 1991 [9], se développe primitivement au niveau de l'hypoderme et réalise une infiltration profonde appelée panniculite ou hypodermite souvent situé aux membres inférieures. Le tableau clinique habituel est celui de placards hypodermiques avec des nodules sous cutanés [10], accompagné parfois de signes généraux notamment à une altération de l'état général avec fièvre en rapport avec un syndrome d'activation macrophagique comme c'est le cas de notre patient. Le lymphome T sous cutané affecte le plus souvent l'adulte jeune avec une légère prédominance féminine [8], contrairement à notre cas, il s'agit d'un adolescent de 16 ans. Deux entités de lymphome T touchant l'hypoderme existe [11], elles se distinguent par des caractéristiques cliniques, histologiques et immunophenotypiques. L'association au SAM est fréquente dans le phénotype CD4- CD8- CD56+, alors que le phénotype CD4- CD8+ CD56- est rarement associé au SAM [4, 12]. Ce qui explique que notre cas (CD4- CD8+ CD56-) est une entité rare survenant chez un garçon d'une tranche d'âge plus jeune. Le lymphome T sous cutané CD4- CD8+ CD56- peut être associé à une atteinte dysimmunitaire qui pourrait être déclenchée par un traitement médicamenteux [13], notre patient avait une pelade depuis l'âge de 6 ans, ce qui est un fort argument d'une dysimmunité. La corticothérapie constitue une excellente arme thérapeutique en matière du SAM, notre patient avait un état général très altéré avec persistance de la fièvre . l'administration des bolus de à base de méthylprednisolone était efficace pour stabiliser son état, puis une chimiothérapie a été démarré dans un service de cancérologie adulte avec bonne évolution.

## Conclusion

Notre observation clinique illustre les difficultés diagnostiques d'un lymphome T sous-cutané révélé par un syndrome d'activation macrophagique et de y penser à cette association malgré le jeune âge.

## Conflits d’intérêts

Les auteurs ne déclarent aucun conflit d'intérêts.
